# Creating customized chatbots with ChatGPT to promote physical activity: a mini review

**DOI:** 10.3389/fdgth.2026.1761193

**Published:** 2026-04-10

**Authors:** Liam O’Malley, Aidan Halley, Amanda Willms, India Edwards, Sam Liu

**Affiliations:** School of Exercise Science, Physical and Health Education, University of Victoria, Victoria, BC, Canada

**Keywords:** AI, AI and healthcare, artificial intelligence, behaviour change, chatbot, ChatGPT, custom*

## Abstract

Artificial intelligence (AI) chatbots powered by large language models (LLMs) such as ChatGPT offer a promising approach for delivering scalable, personalized physical activity interventions. Despite growing interest in applying these tools to health behaviour change, concerns remain regarding accuracy, safety, hallucinations, privacy, and theoretical grounding. This mini-review summarizes current methods for creating customized ChatGPT-based chatbots for physical activity promotion and outlines approaches for evaluating their performance. A literature search was conducted across five databases, white papers, and OpenAI technical reports. Three primary customization strategies were identified: retrieval-augmented generation (RAG), system prompt engineering, and fine-tuning. RAG enhances accuracy by grounding responses in curated guidelines and behaviour-change frameworks. System prompts define the chatbot's role, tone, and reasoning logic. Fine-tuning adapts the model's communication style using expert-crafted prompt–response pairs. These methods can be implemented independently or in combination, depending on intervention goals. Evaluation of customized chatbots requires both intrinsic model-based testing and extrinsic human-centred assessment. Additional considerations include protecting user privacy by avoiding collecting identifiable data, implementing data-minimization safeguards, and managing token-based operational costs associated with ChatGPT systems. Customized ChatGPT chatbots offer substantial potential for advancing physical activity promotion; however, safe and effective deployment requires thoughtful design, rigorous evaluation, and careful attention to privacy and cost.

## 1 Introduction

Artificial intelligence (AI) chatbots are computer-based conversational agents designed to simulate human dialogue by interpreting user input and generating appropriate responses. Modern AI chatbots often rely on Large Language Models (LLMs), which are advanced AI systems trained on vast amounts of text data to understand, generate, and reason with human language. GPT (Generative Pre-trained Transformer) is a specific family of LLMs developed by OpenAI and is built using a deep neural network architecture to produce coherent context-aware responses (1). Recent advancements in GPT models have enabled multimodal interaction, allowing chatbots to interpret text, speech, visual information, and contextual cues. As a result, researchers are increasingly using existing LLM platforms, particularly ChatGPT, to accelerate the development of conversational agents (2).

AI chatbots have shown promise in delivering personalized, scalable behaviour-change interventions across areas such as promoting healthy lifestyles, smoking cessation, treatment or medication adherence, and managing substance use (3). A recent meta-analysis reported that AI chatbot interventions can significantly improve total physical activity (*SMD* = 0.28, *95% CI* = 0.16–0.40), daily steps (*SMD* = 0.28, *95% CI* = 0.17–0.39), and moderate-to-vigorous physical activity (*SMD* = 0.53, *95% CI* = 0.24–0.83) (4). Despite these promising results, concerns remain regarding ChatGPT's accuracy, its susceptibility to hallucinations (i.e., confidently presenting false or misleading information), potential privacy vulnerabilities (5).

To address current limitations, researchers are developing customized chatbots driven by behaviour change theories and frameworks, using domain-specific knowledge libraries [e.g., Multi-Process Action Control (M-PAC) framework] (6). ChatGPT facilitates the creation of customized chatbots with minimal coding or technical skills. In this paper, we summarize methods for creating customized ChatGPT-based chatbots for physical activity promotion and describe current approaches for evaluating their accuracy. We then outline future considerations for designing these systems, including privacy, security, and cost.

## 2 Methods

We conducted a literature search in November 2025 on creating customized chatbots using ChatGPT. We searched relevant search engines and databases (Google Scholar, Journal of Medical Internet Research, Frontiers, Nature Partner Journals Digital Medicine and PubMed). Keywords included AI, ChatGPT, Custom*, AI and Healthcare, Retrieval Augmented Generation, RAG, Fine-Tuning, Prompt, Prompt Engineering. We also searched technical reports, white papers, and OpenAI blog posts.

## 3 Results

### 3.1 Steps for custom GPT development

Developing a customized ChatGPT-based chatbot involves layering system instructions, domain-specific content, and optional training data onto the base GPT model to shape its behaviour without modifying the model's underlying architecture. Currently, a ChatGPT Plus account is needed for creating customized chatbots. After creating and upgrading the account, the researcher can access “explore GPTs” and select “Create” to create a GPT. This will take the user to a blank GPT. There are two tabs at the top of the builder: “Create” and “Configure”. The create tab uses an automatic builder that generates behaviour and instructions based on conversational language provided by the user. The Configure tab enables a more robust setup since it allows for direct control over the “Name”, “Description”, “Instructions”, “Conversation Starters”, “Knowledge”, “Model”, and additional “Capabilities”. Although the customization methods are similar across GPT versions, newer models (e.g., GPT-4, GPT-4.1, GPT-5) generally provide stronger reasoning, improved accuracy, and more reliable adherence to instructions than earlier models such as GPT-3.5 (7). Additionally, earlier models may still be selected when cost or computational efficiency is a priority, as the customization methods are compatible among model versions. In the following sections, we describe the three core methods for customizing ChatGPT-based chatbots [system prompt engineering, retrieval augmented generation (RAG), and fine-tuning] and illustrate how each method can be applied through an example physical activity chatbot.

The example chatbot presented in this work was conceptualized to provide physical activity promotion for people with Type 2 Diabetes (T2D). The underlying theory selected to base this chatbot on was the M-PAC Framework. While several behaviour change theories exist (e.g., the Theory of Planned Behaviour and the Transtheoretical Model), we selected the Multi-Process Action Control (M-PAC) framework because it explicitly addresses the intention–behaviour gap. M-PAC extends traditional social-cognitive models by integrating reflective processes involved in intention formation with regulatory and reflexive processes (e.g., planning, habit, identity) that support translating intentions into sustained behaviour change (6). In addition, our team has previously developed and evaluated several mHealth interventions informed by M-PAC (8, 9), making it a theoretically and practically appropriate framework for this project.

#### 3.1.1 System prompt engineering

System prompt engineering refers to the process of creating instructions that tell an AI system how to think and respond. System prompts are the hidden instructions that guide AI behaviour and can be designed by researchers to define the chatbot's role and how the model interprets and responds to participants. These prompts remain constant across interactions, establishing the model's domain, tone, and style (10). System prompts can be tailored to incorporate specific uploaded knowledge base documents (see Section 3.1.2 RAG), the communication style and tone, and to direct the model. Table 1 shows an example of what system prompts may be needed for a customized chatbot for physical activity promotion in adults with T2D.

**Table 1:** Example system prompts for designing a physical activity promotion chatbot for adults with T2D.

Component/consideration	System prompt example
Who the chatbot is	You are a physical activity coach for adults with T2D, providing guidance that is safe, supportive, and evidence-based.
Which behaviour change framework guides its logic	Use the M-PAC framework to determine how to promote PA. Specifically, use the validated M-PAC questionnaires to assess reflective, regulatory and reflexive processes (31).
How it should behave	Respond in a supportive, non-judgmental tone; prioritize participant safety; encourage gradual progression; and avoid making medical diagnoses or treatment decisions.
Which sources to rely on (explicit RAG instruction)	When generating responses, rely only on the uploaded RAG knowledge base, including T2D physical activity guidelines and M-PAC framework documents.
What to avoid	Do not provide advice that contradicts the uploaded guidelines or appears medically inappropriate, unsafe, or outside the scope of physical activity coaching.

For example, for a physical activity chatbot for adults with T2D, consider a scenario where a research participant enters the message: *“Some days it's hard to stay motivated; are there easy exercises I can start?”* The chatbot first interprets this input through its hidden system prompt instructions. Since the system prompt defines the chatbot as *“a physical activity coach for adults with T2D,”* the model understands that it should provide T2D-specific physical activity guidance rather than generic exercise advice. The system prompt also requires the chatbot to apply the M-PAC framework when interpreting participant messages. In this case, the participant's difficulty maintaining motivation suggests a reflective-process challenge, meaning the participant has formed some intentions and perhaps begun engaging in physical activity. Based on this interpretation, the chatbot may ask brief follow-up questions about exercise opportunities, motivation and self-regulation skills (6).

Since the system prompt instructs the chatbot to draw exclusively from its RAG knowledge base (e.g., T2D physical activity guidelines, M-PAC summaries, behaviour change technique lists), any guidance provided, such as building environmental cues, reinforcing identity statements, or establishing habit-formation strategies, is grounded in verified sources rather than the model's general pretraining. The system rules specifying a supportive tone, clear safety constraints, and avoidance of medically inappropriate advice further shape how the chatbot formulates its response.

A resulting response from ChatGPT with the above system prompts:

“It sounds like you're at a point where being active feels helpful, but it hasn't quite become a steady routine yet. That's completely normal. Many adults with T2D find it easier to stay active when the activity fits naturally into their day, for example, a short walk after meals or a simple stretching routine at the same time each evening. These small patterns can help activity feel more automatic over time. What usually helps you stay on track, even on days when motivation is low?” (11).

#### 3.1.2 Retrieval augmented generation

RAG is a machine learning and natural language processing approach that enhances the performance of LLMs by combining 1) the generative abilities of a model such as ChatGPT, and 2) an external, verified knowledge base that the model can search in real time (12). Traditional LLMs generate responses based solely on patterns learned during pre-training, which can lead to “hallucinations”, where the model produces outputs that seem plausible but are incorrect (12). RAG was developed to address this limitation. Instead of relying only on the model's internal knowledge, RAG retrieves information from curated documents (e.g., clinical guidelines, academic papers, protocols, manuals) and incorporates that information directly into the model's responses (32). This ensures greater accuracy and transparency by grounding the chatbot's output in specific sources.

RAG works by converting uploaded documents into a searchable index. When a research participant asks a question, the customized chatbot conducts a retrieval step before generating an answer. Depending on the nature of the query, the model may use semantic search, which identifies the most relevant document segments based on meaning, or perform a document review, in which the model systematically scans the full set of documents to extract verbatim or highly specific information (13). The customized chatbot then uses these retrieved segments as the basis for its response. In ChatGPT, RAG is implemented by uploading PDF, text, or web content into the Custom GPT's “Knowledge” library, allowing the chatbot to rely exclusively on these sources. This approach is particularly valuable for physical activity interventions as it enables researchers to anchor chatbot responses in verified physical activity guidelines, safety recommendations, and behaviour change frameworks, without retraining or modifying the underlying GPT model.

Building on the earlier example of a physical activity chatbot designed for adults with T2D, the knowledge base may incorporate the M-PAC framework and specific clinical physical activity guidelines for T2D. With this knowledge base in place, when a research participant enters a chatbot message such as, *“Some days it's hard to stay motivated; are there easy exercises I can start?”*, the chatbot can use RAG to retrieve appropriate evidence-based strategies such as coping planning, action planning, or barrier management techniques from the uploaded M-PAC and physical activity guideline documents.

#### 3.1.3 Fine-tuning

Fine-tuning in ChatGPT is a supervised learning method where a separate version of the GPT model is trained on domain-specific datasets to adapt its language style, tone, and content. Unlike RAG and system prompt engineering, fine-tuning using ChatGPT adjusts the parameters of a customized copy of the model. Instead of building a GPT model from scratch, fine-tuning works by giving the model additional examples for the model to learn how the chatbot should communicate. The GPT model already understands language, reasoning, and conversation in a broad general way, so large amounts of data are not needed to train the new model (14). Through fine-tuning, researchers can align the model's responses more closely with the domain-specific knowledge and communication practices reflected in their dataset.

ChatGPT-5.0 supports fine-tuning through OpenAI's Application Programming Interfaces (APIs), which connect a structured training file to the customized model. These training files follow OpenAI's JSON format and contain prompt-response pairs, where each user prompt is paired with an expert-crafted response (15). For our example, a training document might include question-response pairs that illustrate how a health coach or educator would support individuals with T2D across the phases of M-PAC. These pairs could include outcome expectations and perceived opportunity (reflective), strategies for action planning or coping planning when motivation fluctuates (regulatory), and ways to reinforce emerging habits or identity shifts related to being an “active person” (reflexive). To improve generalizability and equity, the dataset can incorporate responses from diverse participant profiles that represent variation in age, gender, cultural background, diabetes management experience, motivation, and lifestyle behaviours (16). Table 2 provides examples of participant prompts and their corresponding responses, illustrating how fine-tuning data can embed supportive tone, diabetes-appropriate physical activity guidance, and M-PAC behavioural processes into the chatbot's communication patterns.

**Table 2:** Example user prompts and expert-crafted responses for fine-tuning a physical activity chatbot for adults with T2D.

User prompt	M-PAC process & considerations	Expert-crafted response (now reflecting diverse profiles)
“I keep losing motivation after a few days.”	Regulatory process; fluctuating motivation common across ages and lifestyles	“It's very common for motivation to rise and fall, especially if you balance work, caregiving, or other responsibilities. One strategy is to plan for days when energy is low. What usually makes it harder for you after a few days? We can create a coping plan that fits your routine and environment.”
“Is it safe for me to start walking if I have T2D?”	Reflective process; safety guidance for individuals with varying health statuses	“For most adults with T2D, beginning with low-intensity walking is safe and beneficial. If you experience foot problems, dizziness, or have limited mobility, we can explore alternatives like seated exercises or short indoor bouts. Start with what feels comfortable and gradually increase as you're ready.”
“I don't feel confident being active outside.”	Reflective process; cultural norms, neighbourhood safety, and weather concerns	“It makes sense to feel unsure—confidence can depend on safety, cultural comfort, and access to supportive spaces. Some people prefer home-based routines, indoor walking videos, or activity at community centres. Which environment feels safest and most comfortable for you to start with?”
“I want to exercise more, but I don't know where to start.”	Reflective & Regulatory; accommodating different cultural, economic, and lifestyle contexts	“Starting small works well for many people. Think about movements that fit your daily life—for example, a short walk after meals, stretching during breaks, or culturally familiar activities like dance. Which options feel realistic for your home, schedule, or preferences?”
“Sometimes I forget to exercise even when I want to.”	Reflexive process; habit-building across diverse routines	“This is very common before activity becomes a habit. We can build cues that fit your lifestyle—like reminders on your phone, pairing activity with prayer times, meal routines, or children's bedtime. What daily routine could we link your activity to?”
“Exercise feels expensive—I can't afford a gym.”	Reflective; socioeconomic constraints	“That's completely understandable. Many effective activities cost nothing—walking, stair climbing, stretching, or simple home routines. We can build a plan using free resources. What free options feel most doable for you?”
“English isn't my first language, so some instructions confuse me.”	Reflective; linguistic and cultural accessibility considerations	“Thank you for sharing that. I can provide simpler explanations or step-by-step guidance. If you prefer certain types of examples or familiar activities, let me know and I'll adjust the way I explain things.”

Overall, these three chatbot customization methods can be used independently or in combination, depending on the goals of the intervention and the resources available. In many cases, layered approaches are most effective. For example, system prompts paired with RAG can enforce behavioural rules while grounding responses in verified documents, whereas system prompts combined with fine-tuning can ensure a consistent tone and strong theoretical alignment. Fine-tuning does not replace system prompts or RAG; rather, it complements them: system prompts set expectations, RAG ensures factual accuracy, and fine-tuning teaches the model how to communicate (17).

### 3.2 Evaluating customized chatbots

Evaluating a customized ChatGPT-based chatbot is essential to ensure that it operates accurately and safely (18). The first method is intrinsic (chatbot model-based) evaluation, which examines the chatbot's internal functioning using automated analyses of chatbot output clarity and relative performance (19). This type of assessment is typically conducted using computational benchmark tests that evaluate the model's performance without involving research participants. These tests may include comparisons with previous model versions, logic assessments, toxicity or harm detection, stress-testing, or checks for hallucinations and guideline violations (20). For a customized chatbot designed to promote physical activity for adults with T2D, intrinsic evaluation could involve prompting the customized chatbot with a wide range of structured scenarios that mirror physical activity counselling conversations. These scenarios may include general questions about diabetes-specific physical activity guidelines, as well as more specific instances such as hypoglycemia, peripheral neuropathy, exercise-induced foot pain, education or management strategies. The chatbot's responses are then analyzed using several computational metrics, including semantic coherence (e.g., the logical structure of responses), grammatical quality, model-to-model comparisons across chatbot iterations, and alignment with gold-standard clinical responses using automated scoring methods such as the Bilingual Evaluation Understudy, BLEU metric (19). When designing chatbots powered by established LLMs like ChatGPT, built-in safety and quality controls screen content and flag potentially risky outputs before deployment. Beyond these controls, ChatGPT currently allows researchers to selectively enable or disable capabilities such as web search, which introduces additional trade-offs relevant to evaluation. Enabling web search may reduce hallucinations on time-sensitive topics but exposes the chatbot to unreliable web content; while disabling it improves consistency at the risk of outdated recommendations, a particularly consequential concern in clinical domains. These configurations and their implications for output accuracy warrant consideration during evaluation. Although these safeguards reduce risk, they do not fully prevent the possibility of harmful or inaccurate outputs.

Intrinsic (model-based) evaluation offers an efficient and scalable way to assess a chatbot's internal functioning, allowing researchers to rapidly identify issues in coherence, fluency, model comparison, and guideline alignment before exposing the system to participants. However, intrinsic methods have several limitations. They cannot determine whether the chatbot accurately reflects theoretical constructs or behaviour change content, or capture user experience (e.g., acceptability, usability). As a result, intrinsic evaluation can be complemented by extrinsic evaluation.

Extrinsic evaluation examines the chatbot's performance in real-world contexts from the perspectives of both researchers and participants (19). Researcher-level assessments focus on aspects such as accuracy, safety, and alignment with evidence-based physical activity and T2D guidelines. The QUEST framework is an established framework which assesses LLM-based chatbots across five dimensions: quality of information, understanding and reasoning, expression style and persona, safety and harm, and trust and confidence (21). Researchers may also engage in human-in-the-loop validation, reviewing and refining chatbot outputs iteratively to ensure accuracy, contextual fit, and safety (22, 23). Recent reviews show substantial variability in how human evaluation is conducted across studies (24). Most studies assess relevance and coherence (e.g., accuracy, comprehensiveness, and reasoning) while far fewer evaluate safety-related harms such as bias, toxicity, hallucinations, or privacy risks. Harm-oriented assessments remain rare, despite being essential for clinical and behavioural applications. Comparisons against human responses or other LLMs are used inconsistently across studies, further contributing to methodological heterogeneity.

At the participant level, evaluation may include usability, acceptability, trust, and perceived empathy. Standardized tools, such as the System Usability Scale (25) assess ease of use but may not capture key conversational qualities of generative-AI agents, such as flow, context retention, or natural language responsiveness (26). Newer measures, such as the Chatbot Usability Scale (BUS-11) (26), address some of these gaps but are not yet validated for adaptive, generative systems. Acceptability assessments often rely on study-specific questions about appropriateness, interpersonal tone, and trustworthiness (27). Importantly, while these intrinsic and extrinsic metrics assess the quality, safety and user experience of chatbot interactions, they do not directly measure whether such interactions translate into sustained physical activity behaviour change. Establishing this link requires long-term outcome evaluation and should not be inferred from conversational performance alone.

## 4 Additional considerations for designing customized chatbots

A major consideration for creating a customized chatbot using ChatGPT is privacy and data protection. Recent evidence suggests that many LLM-based health applications provide vague or incomplete descriptions of deidentification, anonymization, and data-handling practices, or omit consent procedures altogether (28). To avoid these limitations, chatbot-based interventions should clearly document data-security processes across design, implementation, and reporting. Researchers can reduce privacy risks by designing the chatbot to avoid collecting identifiable information altogether. System prompts can instruct the model not to request or retain personal identifiers such as names, addresses, dates of birth, or contact details, and to gently redirect users who attempt to share such information (29). Prompts can also specify that demographic or clinical questions should only be asked when explicitly approved by the study protocol and that all guidance should be delivered without relying on personal identifiers. In addition, applying a data-minimization framework helps prevent inadvertent collection of identifiable data (e.g., exact age, workplace, GPS data, photos). For studies that require storing transcripts, server-side preprocessing tools can be used to filter out identifiers before the data enters research databases (28).

The cost associated with using a customized chatbot is another factor to consider for researchers, as expenses vary widely depending on design and usage. ChatGPT bill usage is based on tokens, which are small units of text (approximately 3–4 characters or 0.75 words each) that represent both the input sent to the model and the output it generates. Every component of a customized chatbot, including system prompts, retrieved RAG documents, user messages, and chatbot responses, all contribute to the total token count for each interaction. This means that longer system instructions (e.g., M-PAC information documents), extensive RAG retrieval, or lengthy participant-chatbot conversations increase operational costs. At the time of this publication, the paid ChatGPT plans include a monthly free token allowance, allowing researchers to prototype early versions of their chatbot at no additional cost. Once the free tokens are used, API pricing for GPT models typically ranges from USD $0.002–$0.02 per 1,000 tokens for smaller models (e.g., GPT-3.5 variants) and USD $0.06–$0.15 per 1,000 tokens for larger, more capable models such as GPT-4.1 or GPT-5. For a moderate-scale research study (e.g., 50–200 participants interacting with a chatbot over several weeks), total API costs may fall between USD $200–$2,000, depending on conversation length and model selection (30). More advanced customization methods, such as fine-tuning and hosting a separate tuned model, can incur additional costs. Fine-tuning requires paying for training compute (USD $50–$500+), depending on dataset size and higher ongoing inference costs that scale with the fine-tuned model. Projects requiring secure, institution-based hosting or integration with protected health information environments may face additional infrastructure and maintenance costs. OpenAI's published pricing for API usage, fine-tuning, and enterprise plans provides researchers with a practical starting point for budgeting (30).

## 5 Discussion and conclusion

Customized ChatGPT-based chatbots offer a promising and accessible way to deliver personalized and scalable physical activity interventions. Researchers can create customized chatbots by applying methods such as RAG, system prompt engineering, and fine-tuning. To ensure these systems operate safely and effectively, researchers should rigorously evaluate them using both intrinsic model-based testing and human-centered extrinsic assessment. These complementary approaches help confirm accuracy, safety, usability, and alignment with behaviour change principles, although the field still lacks standardized and validated evaluation frameworks. Researchers must also consider privacy and data-protection safeguards, including strategies to prevent the collection of sensitive information. Finally, the cost of developing and operating customized chatbots should be considered during study planning and evaluation.
